# Nebulized in-line endotracheal dornase alfa and albuterol administered to mechanically ventilated COVID-19 patients: a case series

**DOI:** 10.1186/s10020-020-00215-w

**Published:** 2020-09-29

**Authors:** Andrew G. Weber, Alice S. Chau, Mikala Egeblad, Betsy J. Barnes, Tobias Janowitz

**Affiliations:** 1 Division of Pulmonary, Critical Care, and Sleep Medicine, Department of Medicine, Northwell Health, 300 Community Drive, Manhasset, NY 11030 USA; 2 Division of Allergy and Infectious Diseases, Department of Medicine, University of Washington and the Center for Immunity and Immunotherapies, Seattle Children’s Research Institute, 1900 9th Ave, Seattle, WA 98101 USA; 3 Cancer Center, Cold Spring Harbor Laboratory, 1 Bungtown Road, Cold Spring Harbor, NY 11724 USA; 4 Center for Autoimmune, Musculoskeletal and Hematopoietic Diseases, The Feinstein Institutes for Medical Research and the Departments of Molecular Medicine and Pediatrics, Donald and Barbara Zucker School of Medicine at Hofstra/Northwell, 350 Community Drive, Manhasset, NY 11030 USA; 5 Northwell Health Cancer Institute, 450 Lakeville Road, New Hyde Park, NY 11042 USA

**Keywords:** SARS-CoV-2, COVID-19, Coronavirus, Mucopurulent secretions, Dornase alfa, Neutrophil extracellular traps, ARDS, VV-ECMO

## Abstract

**Background:**

Mechanically ventilated patients with COVID-19 have a mortality of 24–53%, in part due to distal mucopurulent secretions interfering with ventilation. DNA from neutrophil extracellular traps (NETs) contribute to the viscosity of mucopurulent secretions and NETs are found in the serum of COVID-19 patients. Dornase alfa is recombinant human DNase 1 and is used to digest DNA in mucoid sputum. Here, we report a single-center case series where dornase alfa was co-administered with albuterol through an in-line nebulizer system.

**Methods:**

Demographic and clinical data were collected from the electronic medical records of five mechanically ventilated patients with COVID-19—including three requiring veno-venous extracorporeal membrane oxygenation—treated with nebulized in-line endotracheal dornase alfa and albuterol, between March 31 and April 24, 2020. Data on tolerability and response were analyzed.

**Results:**

The fraction of inspired oxygen requirements was reduced for all five patients after initiating dornase alfa administration. All patients were successfully extubated, discharged from hospital and remain alive. No drug-associated toxicities were identified.

**Conclusions:**

Results suggest that dornase alfa will be well-tolerated by patients with severe COVID-19. Clinical trials are required to formally test the dosing, safety, and efficacy of dornase alfa in COVID-19, and several have been recently registered.

## Background

Critically ill patients with coronavirus disease 2019 (COVID-19), caused by the severe acute respiratory syndrome coronavirus 2 (SARS-CoV-2), progress to hypoxemic and then mixed respiratory failure, secondary to acute respiratory distress syndrome (ARDS) (Marini and Gattinoni 2020; Greenland et al. 2020). Approximately 79–88% of patients admitted to the intensive care unit (ICU) with COVID-19 require intubation and mechanical ventilation, with a mortality of 24–53% (Cummings et al. 2020; Grasselli et al. 2020; Richardson et al. 2020; Docherty et al. 2020). ARDS in COVID-19 is characterized by respiratory failure, in part attributable to distally located mucopurulent secretions.

Dornase alfa (Pulmozyme®) is recombinant human DNase 1 and a safe mucolytic that is administered in nebulized form. It is FDA-approved in combination with standard therapies for patients with cystic fibrosis to improve sputum clearance and pulmonary function (Yang and Montgomery 2018). It is also used off-label as a mucolytic in other diseases, including ARDS (Morris and Mullan 2004; ClinicalTrials.gov [Internet] 2019). A mechanism by which dornase alfa might improve ventilation is by reducing the DNA-mediated viscosity of neutrophil-rich secretions (Papayannopoulos et al. 2011). There are multiple sources for the DNA in mucoid sputum, one of which is neutrophil extracellular traps (NETs). Recently, we collaboratively reported that in the discarded serum of patients with COVID-19, the levels of NETs were increased and correlated with lactate dehydrogenase (LDH), D-dimer, and C-reactive protein (CRP) levels (Zuo et al. 2020). Subsequently, NET-containing microthrombi and increased neutrophil-platelet infiltration in pulmonary autopsies from COVID-19 patients was reported (Becker 2020; Fox et al. 2020; Middleton et al. 2020; Schonrich et al. 2020; Tay et al. 2020; Varga et al. 2020; Leppkes et al. 2020). Notably, targeting NETs reduces mortality in animal models of acute lung injury (Barnes et al. 2020; Adrover et al. 2020; Caudrillier et al. 2012; Lefrancais et al. 2018; Thomas et al. 2012). Despite recognition that mucolytic treatment may be beneficial for patients with COVID-19 (12, (Earhart et al. 2020), administration of nebulized medications, such as dornase alfa, have been limited due to risk of viral aerosolization. If risk of viral aerosolization can be avoided, dornase alfa may benefit patients with severe COVID-19 by acting as a mucolytic and by reducing NET levels in the lungs, thereby improving oxygenation and ventilation. We report the clinical course, safety, and outcomes after nebulized in-line endotracheal dornase alfa treatment for five intubated and mechanically ventilated patients with PCR-confirmed COVID-19.

## Methods

The Northwell Health institutional review board that focuses on COVID-19 research approved this case series as minimal-risk research using de-identified data from routine clinical practice. Data were collected from the enterprise health record (Sunrise Clinical Manager; Allscripts) reporting database, and included patient demographics, comorbidities, inpatient medications, laboratory studies, treatment, and outcomes. We further obtained longitudinal values of the fraction of inspired oxygen (FiO_2_) and of the arterial partial pressure of carbon dioxide (PaCO_2_) as measures of respiratory function during treatment. Lung mechanics were assessed by obtaining mechanical power of the respiratory system (MP) and mean airway pressure (Paw). FiO_2_ values of the circuit were reported for those patients who required veno-venous extracorporeal membrane oxygenation (VV-ECMO). Ferritin, CRP, LDH, and D-dimer were obtained as measures of systemic disease and inflammation. Not all patients had laboratory investigations on the same days in relation to the nebulized and co-administered dornase alfa and albuterol (nDA + A) treatment. In the following case synopses, each measurement is therefore followed by the day in relation to the first day of treatment with nDA + A (e.g.*,* d 2 for the second day of treatment with nDA + A or d − 1 for the day before nDA + A treatment was initiated). Relevant nDA toxicities (hoarseness, rash, hypersensitivity reactions and hemoptysis) were clinically monitored. Hoarseness was assessed post-intubation. Rash was assessed daily via physical exam. Hypersensitivity reactions were determined via physical exam, vital signs, and vasopressor requirements. Hemoptysis was assessed in intubated patients by endotracheal tube visual inspection.

## Results

Five patients treated with dornase alfa between March 31, 2020 and April 24, 2020 were identified. These patients had met the Berlin criteria for ARDS and were treated with ventilator strategies guided by the ARDSNet protocol at North Shore University Hospital within Northwell Health (Durante et al. 2002). These patients had been treated with dornase alfa because they required high levels of FiO_2_ and had elevated ventilation demands. All patients received the same treatment doses: nebulized dornase alfa (2.5 mg) co-administered twice daily with the short-acting β_2_-agonist albuterol (2.5 mg) to improve delivery to the alveoli (referred to as nDA + A). Of note, β_2_-adrenoreceptor agonism may also inhibit NET formation by direct action on neutrophils (Marino et al. 2018). The treatment was administered with an Aerogen® Solo in-line nebulizer to avoid open aerosol generation, which would place staff at risk of exposure to SARS-CoV-2.

**Fig. 1:**
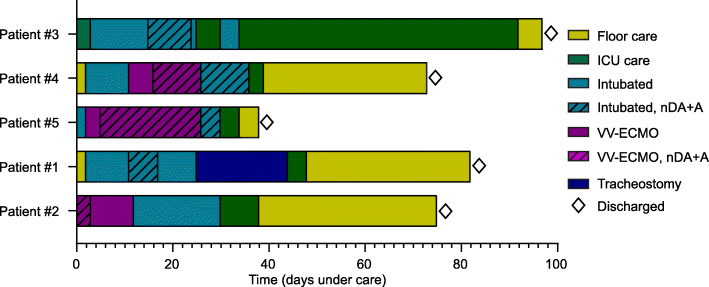
Overview of the clinical course of five patients treated with nebulized dornase alfa + albuterol (nDA + A)

**Fig. 2:**
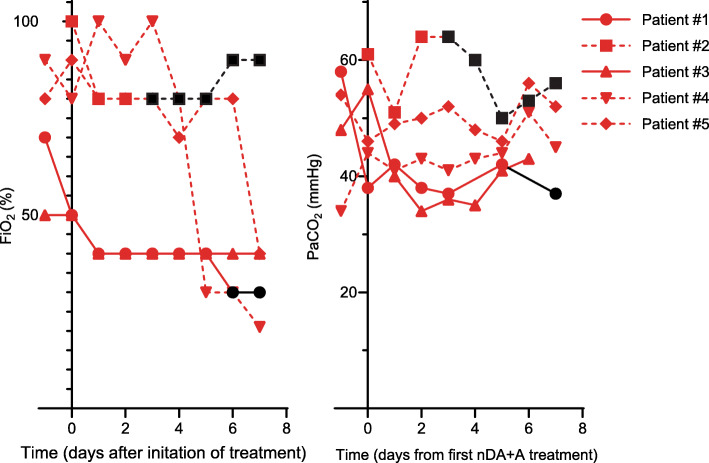
Patient-level data of respiratory function during treatment with nebulized dornase alfa + albuterol (nDA + A). Values were extracted from the medical records the day before and up to the seven days after the initiation of treatment. Values are graphed in black for patients after they ceased nDA + A treatment. Dashed lines indicate patients on VV-ECMO. Not all markers were measured daily for every patient. FiO_2_: fraction of inspired oxygen; PaCO_2_: partial pressure of carbon dioxide

**Fig. 3:**
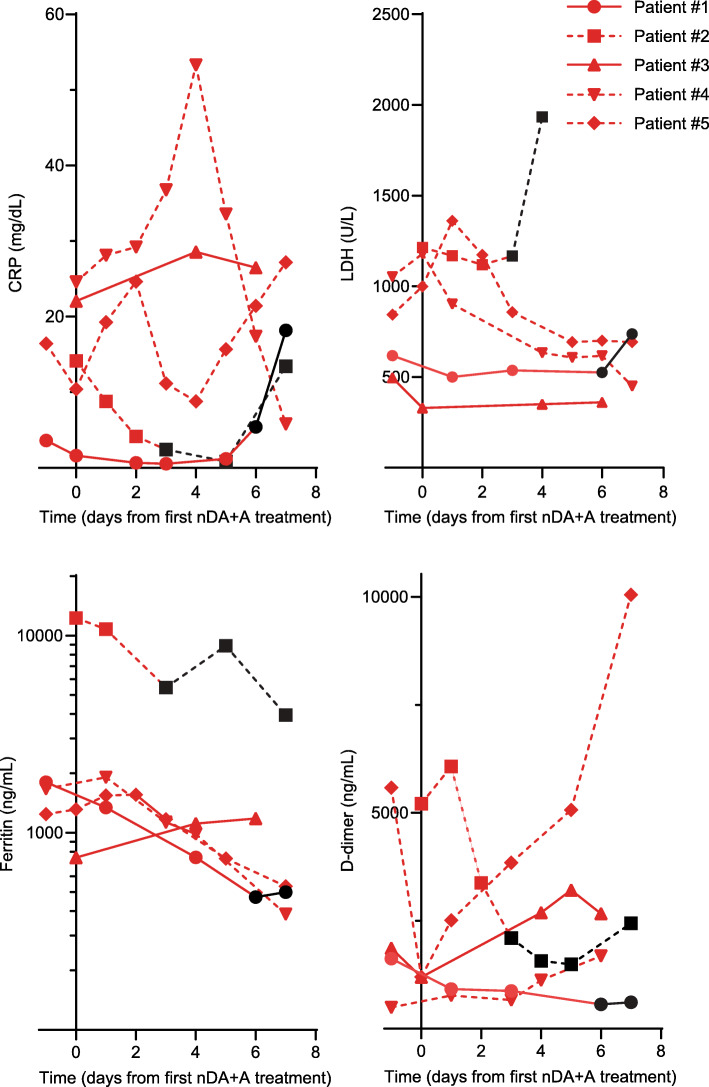
Patient-level data of systemic disease during treatment with nebulized dornase alfa + albuterol (nDA + A). Values were extracted from the medical records the day before and up to the seven days after the initiation of treatment. Values are graphed in black for patients after they ceased nDA + A treatment. Dashed lines indicate patients on VV-ECMO. Not all markers were measured daily for every patient. CRP: C-reactive protein; LDH: lactate dehydrogenase

The patient characteristics are summarized in Table 1. Patients were treated with nDA + A between 3 to 25 days. The most common characteristics of the patients included obesity (BMI ≥ 30) and four of the patients had hypertension. Four patients received methylprednisolone dosed at 1-2 mg/kg/day. All patients were treated with full dose or prophylactic dose anticoagulation for thrombosis. All other medications that were administered during the course of hospitalization were considered standard of care (STC) at the time of treatment and are summarized in Table S1. No adverse events related to nDA + A treatment were observed for any of the patients throughout the duration of the study. The clinical course of the five patients treated with nDA + A is summarized in Fig. 1. Figures 2 and 3 display the longitudinal, ventilatory, and inflammatory markers for each patient.

**Table 1:** Patient data from five patients with COVID-19 who received dornase alfa with albuterol March–April, 2020

Patient	1	2	3	4	5
Clinical Characteristics					
Date of admission	29 March	4 April	16 March	16 March	26 March
Age	56	34	65	31	34
Gender	F	M	M	M	F
Ethnicity	Hispanic	White	Asian	Hispanic	Black
BMI	38	41	32	30	38
Date of ICU admission	31 March	4 April	16 March	18 March	26 March
Comorbidities					
Hypertension	Yes	Yes	Yes	Yes	
Diabetes mellitus, type 2	Yes				
Asthma	Yes		Yes		Yes
Hyperlipidemia		Yes			
Migraine					Yes
Chronic gastritis					Yes
ECMO					
Date of ECMO initiation	–	4 April	–	27 March	28 March
Date of ECMO cessation	–	16 April	–	10 April	20 April
Dornase alfa (DA) + albuterol (A) parameters					
Administration (DA: 2.5 mg, A: 2.5 mg, both twice daily using the Aerogen® Solo nebulizer)					
Date of DA + A initiation	9 April	4 April	31 March	1 April	31 March
Date of DA + A cessation	14 April	6 April	8 April	19 April	24 April
Toxicities	None	None	None	None	None
Other COVID-19 treatment					
Methylprednisolone	Yes	Yes		Yes	Yes
Anakinra		Yes		Yes	
CytoSorb					Yes
Anticoagulants ^a^					
Enoxaparin	40 mg BID	120 mg BID	40 mg BID	100 mg BID	120 mg BID
Argatroban		Yes		Yes	Yes
Heparin gtt			Yes		Yes
Venous thromboembolism	None	None	None	Right SDVT Right CVT	None
Current State	ICU discharge	ICU discharge	ICU discharge	ICU discharge	ICU discharge

^a^Patients were not on simultaneous anticoagulation therapies. *BMI* Body mass index, *ICU* Intensive care unit, *ECMO* Extracorporeal membrane oxygenation, *BID* Bis in die (twice a day), *gtt* Guttae (intravenous drip), *SDVT* Soleal deep vein thrombosis, *CVT* Cephalic vein thrombosis

Patient 1 is a 56-year-old Hispanic woman who presented in respiratory distress. Her respiratory status deteriorated over 48 h, requiring intubation and transfer to the ICU. She was treated with nDA + A for 6 days, starting from day 10 of intubation. The FiO_2_ requirement decreased from 70% (d − 1) to 30% (d 6), PaCO_2_ from 58 (d − 1) to 37 mmHg (d 7), ferritin from 1803 (d − 1) to 472 ng/mL (d 6), and D-dimer from 1619 (d − 1) to 563 ng/mL (d 6). MP had increased from 16.48 (d − 1) to 20.91 J/minute (d 6), and Paw from 12 (d − 1) to 20 cmH_2_O (d 6). Minimal changes were noted in CRP and LDH. The patient underwent a tracheostomy after 23 days of endotracheal intubation and was able to be decannulated 19 days later. She was discharged to a rehabilitation facility after a total hospital stay of 82 days.

Patient 2 is a 34-year-old white man who presented to the hospital in diabetic ketoacidosis without prior history of diabetes mellitus. He was intubated on admission and initiated on VV-ECMO. He received nDA + A for 3 days and was de-cannulated after 12 days. No change in ECMO settings occurred during treatment time. The FiO_2_ requirement decreased from 100% (d 0) to 80% (d 3), MP from 36.38 (d 0) to 12.03 J/min (d 3), Paw from 36.38 (d 0) to 12.03 cmH_2_O (d 3), CRP from 14.14 (d 0) to 2.41 mg/dL (d 3), ferritin from 12,281 (d 0) to 5453 ng/mL (d 3), and D-dimer from 5210 (d 0) to 2099 ng/mL (d 3). Minimal changes were noted in PaCO_2_ and LDH. The patient was able to be extubated after 30 days of endotracheal intubation (VV-ECMO the first 12 days). He was discharged to a rehabilitation facility after a total hospital stay of 75 days.

Patient 3 is a 65-year-old Asian man who was admitted directly to the ICU for respiratory distress and intubated 3 days later. Twelve days after intubation, he was started on 9 days of nDA + A treatment. The FiO_2_ requirement decreased from 50% (d − 1) to 40% (d 7), PaCO_2_ from 55 (d 0) to 43 mmHg (d 6), and CRP from 22.07 (d 0) to 26.48 mg/dL (d 6). Minimal changes were noted in MP, Paw, ferritin, LDH, and D-dimer. He was extubated 1 day after the completion of the nDA + A course. Five days later, he was re-intubated for an additional 4 days due to mental status changes and failure to protect his airway. The patient was extubated in ICU care 30 days after his initial endotracheal intubation. He subsequently developed a right colonic and small bowel perforation requiring the placement of multiple drains. He required the initiation of total parenteral nutrition until he was able to tolerate enteral feeds. Surgery was never required. He was discharged to a rehabilitation facility after a total hospital stay of 97 days.

Patient 4 is a 31-year-old Hispanic man who was intubated and transferred to the ICU from the Internal Medicine service 2 days after presenting with respiratory distress. Nine days after intubation, he was initiated on VV-ECMO. Five days after cannulation, he was started on the nDA + A treatment. After ten days, he was de-cannulated and remained intubated for ten days while continuing the nDA + A treatment. No change in ECMO settings occurred during treatment time. He was then extubated and discharged to the floor. The FiO_2_ requirement decreased from 90% (d − 1) to 21% (d 7), Paw from 19 (d − 1) to 16 cmH_2_O (d 7), and LDH from 1054 (d − 1) to 451 U/L (d 7). Ferritin initially decreased from 1669 (d − 1) to 387 ng/mL (d 7). On day 15 of treatment, he developed methicillin-resistant *Staphylococcus aureus* (MRSA) pneumonia and bacteremia. Ferritin thus increased to 1619 ng/mL (d 13) prior to decreasing to 555 ng/mL (d 19) with antibiotic treatment. Minimal changes were noted in MP, PaCO_2_, CRP, and D-dimer. He was transferred to an acute inpatient rehabilitation facility after a total hospital stay of 73 days.

Patient 5 is a 34-year-old black woman who was intubated at an outside hospital, then transferred to the North Shore University Hospital ICU. Two days later, she was cannulated for VV-ECMO. She required VV-ECMO for 24 days and was intubated for a total of 30 days. She was treated with nDA + A for 25 days starting on day 4 following cannulation for VV-ECMO. While on VV-ECMO for the first 5 days, CytoSorb therapy was applied. She was de-cannulated after 24 days, extubated after 4 days, and discharged to the floor. No change in ECMO settings occurred during treatment time. The FiO_2_ requirement fell from 80% (d − 1) to 40% (d 7), ferritin from 1244 (d − 1) to 535 ng/mL (d 7), and LDH from 844 (d − 1) to 693 U/L (d 7). MP increased from 11.03 (d − 1) to 20.08 J/minute (d 7), Paw from 15 (d − 1) to 22 cmH_2_O (d 7). Minimal changes were noted in PaCO_2_, CRP, and D-dimer. She declined rehabilitation therapy and was discharged home after a total hospital stay of 38 days.

## Discussion

At the doses utilized, no nDA + A treatment-associated toxicities were identified. FiO_2_ requirements decreased for all five patients 7 days after nDA + A treatment was initiated, while measures of lung mechanics varied. All patients were discharged from the hospital and remain alive at the time of submission of this report. During the time period that these patients were treated, ~ 25% mortality was reported during the first month for patients requiring ICU care within our hospital system (Richardson et al. 2020). We recognize that changes in FiO_2_ levels, lung mechanics, and systemic inflammatory markers may be independent of the nDA + A treatment. Clinical trials are therefore required to test the dose range, safety, and efficacy of dornase alfa in patients with COVID-19 in this setting and possibly earlier in the disease course. Endpoints should include measurements of the effect on respiratory function and mechanics, as well as on systemic inflammation, coagulopathy, secondary infections, and the presence of NETs in plasma. Eight such trials were recently registered (*NCT04359654, NCT04355364, NCT04432987, NCT04409925, NCT04445285, NCT04402944, NCT04402970, NCT04459325).*

Although it is not yet clear whether nebulized dornase alfa will have any effect on blood NET levels or systemic inflammation in COVID-19, a reduction in systemic inflammatory markers has been reported after use of dornase alfa in patients with cystic fibrosis (Yang and Montgomery 2018). We did note a reduction in CRP in two patients (patients 2 and 3) and a reduction in D-dimer in two patients (patients 1 and 2) during nDA + A treatment. LDH was reduced for the patients on VV-ECMO during nDA + A treatment, and ferritin was reduced in four out of five patients. Due to the small sample size and the common occurrence of secondary infections in ventilated patients with COVID-19, we are unable to comment on any potential relationship between nDA + A administration and the risk of secondary infections.

## Conclusions

This case report suggests that nebulized dornase alfa in combination with albuterol is a safe treatment option, supporting randomized, controlled clinical trials for mechanically ventilated patients with ARDS secondary to COVID-19, including for those on VV-ECMO—a patient population with an urgent, unmet need for effective therapies.

## Supplementary information

**Additional file 1 Supplemental Table 1.** Additional medications that dornase alfa+albuterol-treated COVID-19 patients received while in the hospital.

## Data Availability

All data generated or analyzed during this study are included within the article.

## Abbreviations

ARDS: Acute respiratory distress syndrome
BID: Bis in die (twice daily)
COVID-19: Coronavirus disease 2019
CRP: C-reactive protein
d: Day
FiO_2_: Fraction of inspired oxygen
gtt: Guttae (intravenous drip)
ICU: Intensive care unit
LDH: Lactate dehydrogenase
MRSA: Methicillin-resistant *Staphylococcus aureus*
nDA + A: Nebulized dornase alfa plus albuterol
NETs: Neutrophil extracellular traps
PaCO_2_: Arterial partial pressure of carbon dioxide
SARS-CoV-19: Severe acute respiratory syndrome coronavirus 2
VV-ECMO: Veno-venous extracorporeal membrane oxygenation
