# Supplementary material for: Nebulized in-line endotracheal dornase alfa and albuterol administered to mechanically ventilated COVID-19 patients: a case series
Source: Mol Med. 2020 Sep 29;26:91. doi: 10.1186/s10020-020-00215-w (PMC7522910; doi:10.1186/s10020-020-00215-w)
Supplement: Supplementary file 1 — Additional file 1 Supplemental Table 1. Additional medications that dornase alfa+albuterol-treated COVID-19 patients received while in the hospital. [file 10020_2020_215_MOESM1_ESM.docx]

**Supplemental Table 1. Additional medications that dornase alfa+albuterol-treated COVID-19 patients received while in the hospital.**

| Patient | 1 | 2 | 3 | 4 | 5 |
| --- | --- | --- | --- | --- | --- |
| Hospital medications  Amiodarone  Ampicillin  Ascorbic acid  Azithromycin  Bumetanide  Caspofungin  Cefepime  Ceftriaxone  Cisatracurium  Dexmedetomidine  Dobutamine  Esmolol  Fentanyl  Fluconazole  Fosphenytoin  Furosemide  HCQ/CQ  Hydromorphone  Insulin  IVIG  Ketamine  Levetiracetam  Meropenem  Metronidazole  Midazolam  Milrinone  Nicardipine  Nitroprusside  Norepinephrine  Pantoprazole  Phenylephrine  Propofol  Rocuronium  Sodium bicarbonate  TPN  Vancomycin  Vasopressin  Vecuronium  Zosyn | Yes  Yes  Yes  Yes  Yes  Yes  Yes  Yes  Yes  Yes  Yes  Yes  Yes  Yes  Yes  Yes  Yes  Yes | Yes  Yes  Yes  Yes  Yes  Yes  Yes  Yes  Yes  Yes  Yes  Yes  Yes  Yes  Yes  Yes  Yes | Yes  Yes  Yes  Yes  Yes  Yes  Yes  Yes  Yes  Yes  Yes  Yes  Yes  Yes  Yes  Yes  Yes  Yes  Yes  Yes  Yes  Yes | Yes  Yes  Yes  Yes  Yes  Yes  Yes  Yes  Yes  Yes  Yes  Yes  Yes  Yes  Yes  Yes  Yes  Yes  Yes  Yes  Yes  Yes  Yes  Yes  Yes  Yes  Yes  Yes | Yes  Yes  Yes  Yes  Yes  Yes  Yes  Yes  Yes  Yes  Yes  Yes  Yes  Yes  Yes  Yes  Yes  Yes  Yes  Yes |

Note: All antimicrobials were given at treatment doses. HCQ: hydroxychloroquine; CQ: chloroquine; IVIG: intravenous immunoglobulin; TPN: total parenteral nutrition
